# Cholesterol deficiency carriers have lowered serum cholesterol and perform well at an elite cattle show

**DOI:** 10.3168/jdsc.2020-18587

**Published:** 2020-09-02

**Authors:** B.L. Basiel, A.L. Macrina, C.D. Dechow

**Affiliations:** Department of Animal Science, Pennsylvania State University, University Park 16802

## Abstract

Cholesterol deficiency (CD) in Holstein cattle results from an insertion in the *APOB* gene, which traces to the bull Maughlin Storm and was spread to elite show cattle largely through his grandson Braedale Goldwyn. Calves that are homozygous for CD can neither transport dietary fat from the gut nor synthesize low-density lipoproteins, and they perish before 6 mo of age. The mutation may have a partially dominant effect, with lowered cholesterol in heterozygous animals. Our aims were to evaluate serum cholesterol in heterozygous animals, evaluate daily phenotypic records of lactating CD carriers, and determine whether CD carrier status is associated with show ring success. Blood was collected on a single date in May 2018 from 12 carriers and 14 controls and 1 yr later from 17 carriers (7 repeated for 2018) and 27 controls (6 repeated from 2018); animals ranged in age from 14 to 84 mo and varied in stage of lactation and pregnancy status. Serum samples were analyzed for cholesterol concentration (mg/dL) and results were analyzed using mixed models that included linear and quadratic effects of days in milk and days carried calf. Daily phenotypic records of milk yield, milking time, milk conductivity, activity, and body weight were compared between carriers and controls. Additionally, the CD carrier status of cows that were sired by known CD carriers and had available genotypes was recorded. Cows placing in the top 5 and top 10 of their classes at World Dairy Expo between the years of 2006 and 2019 were included in our analysis. Chi-squared tests for equal proportions were evaluated to determine whether the following were associated with CD status: placing in the top 10 with repeated placings from the same cow included, placing in the top 5 with repeated placings, top 10 cows considering individuals only once, and top 5 cows considering individuals only once. Control animals (noncarriers) had significantly higher serum cholesterol (116.21 ± 6.45 mg/dL) than CD carriers (90.15 ± 7.49 mg/dL). There was no difference between carriers and controls in milk yield, milk conductivity, or activity. However, CD carriers had shorter milking times and lower body weights than their herdmates. Further, there were more CD carriers in the top 5 and more carriers tended to place in the top 10 at World Dairy Expo when repeated placings were included. These data support observations that CD is partially dominant and indicate that CD carriers display other phenotypic differences from their herdmates that may give them a small advantage at elite cattle shows.

## Highlights

• Cholesterol deficiency carriers had lower serum cholesterol than noncarriers.
• Cholesterol deficiency carriers had faster milking speeds than noncarriers.
• Cholesterol deficiency carriers had lower body weights than noncarriers.
• Cows that carry cholesterol deficiency tend to have an advantage in the show ring.

## Graphical Abstract

**Figure:**
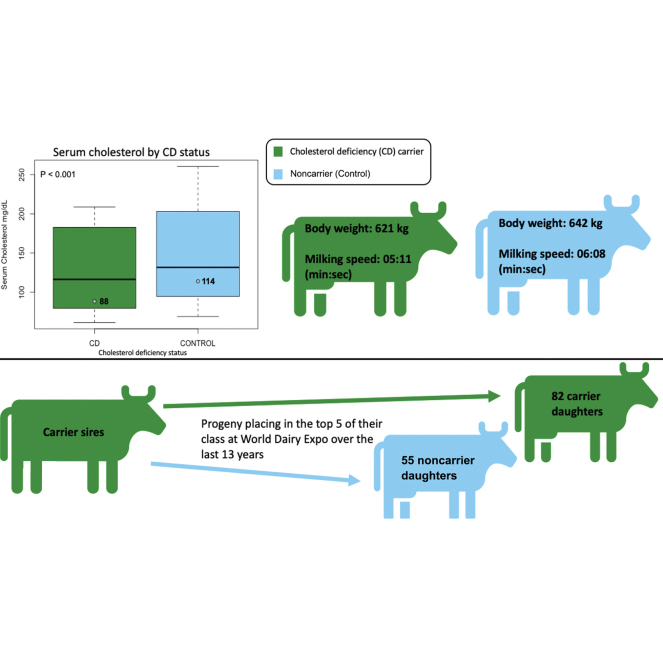
**Summary:** In a small sample of carriers of cholesterol deficiency, the genetic disorder that affects low density lipoprotein transportation in Holstein cattle, serum cholesterol was lower than that of their herdmates. Furthermore, cows that inherited the cholesterol deficiency mutation from their sire placed in the top 5 of their classes at World Dairy Expo more often than their noncarrier paternal sisters.

Cholesterol deficiency (**CD**) is a metabolic disorder that causes low blood cholesterol, reduced feed intake, reduced growth, chronic diarrhea, pneumonia, and eventual death in Holstein calves (Kipp et al., 2016). The CD mutation is caused by an insertion in exon 5 of the *APOB* gene on BTA 11 that results in the production of a nonfunctional, truncated version of apolipoprotein (**APO**) B; the mutation has been traced to the bull Maughlin Storm (0073HO02012) (Menzi et al., 2016; Schütz et al., 2016). Animals affected by CD cannot absorb dietary lipids or fat-soluble vitamins due to the inability to form the APOB 48 isoform in the intestine, and they are incapable of synthesizing low-density lipoproteins because of the inability to form the APOB 100 isoform in liver (Kane, 1983; Whitfield et al., 2004; Gross et al., 2019).

At the time of discovery, the causative mutation in *APOB* was not detectible by standard chips now used for genomic testing, so a haplotype associated with CD (**HCD**) served as the marker. Most genomic tests now directly include the relevant SNP. In the United States Holstein population, the haplotype frequency is estimated to be 2.5% (Cole et al., 2016). The frequency is likely higher in elite show cattle because of the influence of a Maughlin Storm grandson, Braedale Goldwyn (200HO03205), who has been the Premier Sire at World Dairy Expo (https://worlddairyexpo.com/) a record 11 times.

In CD carriers, total blood cholesterol appears to be reduced compared with that of noncarriers, indicating that the mechanism of inheritance of CD is partially dominant (Gross et al., 2016; Kipp et al., 2016; Saleem et al., 2016; Gross et al., 2019; Jacinto et al., 2019). Recently, Häfliger et al. (2019) determined that some CD carriers become symptomatic as calves, like homozygous CD animals.

Cole et al. (2016) determined that CD carriers have more favorable genetic merit than noncarrier Holsteins in national evaluations for the traits fat yield, protein yield, SCS, productive life, daughter pregnancy rate, cow conception rate, and heifer conception rate. However, these differences were marginal so it is unknown whether the differences are biologically relevant. In another study, milk and component yields were found to be unaffected by carrier status (Gross et al., 2019). The only other phenotypic results published on CD carriers determined that fresh and post-thaw quality of sperm from CD carrier bulls used in conventional AI programs did not differ from noncarrier bulls, despite the roles that cholesterol plays in semen quality (Saleem et al., 2016).

Our objectives were to evaluate serum cholesterol levels in CD carriers compared with control herdmates; to investigate differences between mature CD carriers and their noncarrier herdmates in milk yield, milking speed, milk conductivity, activity, and BW; and to determine whether CD carriers have a competitive advantage in the show ring compared with their noncarrier paternal siblings.

Genotyped cows and heifers (IACUC protocol #47580) from the Pennsylvania State University Dairy Teaching and Research Center herd (University Park) were candidates for this study. All animals with an HCD status of 1 (haplotype carrier confirmed by pedigree; n = 3), HCD status of 3 (suspected carrier that could not be confirmed by pedigree; n = 2), and with CD status (confirmed presence of mutation; n = 17) were considered CD carriers. Samples from nulliparous (n = 27), primiparous (n = 20), and multiparous animals (n = 23) were included in our data. Carriers were matched with noncarrier controls by age, stage of lactation, and reproductive status.

In May 2018, blood was collected from 12 CD carriers and 14 noncarrier controls. Blood was again taken in May 2019. Carriers sampled in 2018 that were still in herd were sampled (n = 7), as were controls (n = 6). Additional CD carriers were identified in the herd (n = 10) and controls were age matched (n = 21); we sampled more controls in 2019 to increase the power to detect differences. Samples were from 10 nulliparous CD carriers (age = 21 ± 5 mo) and 17 controls (age = 20 ± 4 mo). Average DIM for lactating cows at the date of sampling was 90 ± 68 (19 CD carriers) and 101 ± 85 (24 controls). The average days carried calf (**DCC**) of pregnant animals was 123 ± 94 (14 CD carriers) and 116 ± 88 (21 controls).

Tubes were stored on ice and centrifuged for 10 min at 1,200 × *g* within 2 h of collection. Serum samples (n = 70) were stored at −20°C and then thawed and analyzed for cholesterol concentration (mg/dL) using an EzyChrom Cholesterol Assay Kit (BioAssay Systems, Hayward, CA) per the manufacturer's instructions.

Daily milk records and BW were compiled over all lactations during the lifetime of CD carriers (n = 13) and controls (n = 19) that had cholesterol levels analyzed and that had calved. Daily milk weight (kg), milking speed, and milk conductivity were recorded at each milking via an automated management system (Afimilk; Kibbutz Afikim, Israel); daily BW were collected by the AfiFarm 3.04E scale system when exiting the milking parlor (S. A. E. Afikim, Rehovot, Israel), and activity levels were determined by a pedometer that also served as electronic identification in the milking parlor. Any records missing both milk yield and BW were eliminated from the set. A total of 18,738 observations from calving to 305 DIM were included in our final analysis: 8,306 observations of CD carriers and 10,432 observations of controls.

Show results were accessed from online records available on World Dairy Expo's website (https://worlddairyexpo.com/pages/Dairy-Cattle-Show-All-Results.php). Lactating Holstein cows that placed in the top 10 in the open show from 2006 to 2019 were considered elite show cows. The CD status of their sires was determined using public records available from Holstein Association USA (HUSA; Brattleboro, VT) or the Canadian Dairy Network (CDN; Guelph, ON, Canada). A total of 13 sires had daughters with available genotypes from HUSA or CDN with daughters in the top 10 of their respective classes: Maughlin Storm, Braedale Goldwyn, Golden-Oaks St Alexander (007HO08221), Lirr Drew Dempsey (007HO09264), Scientific Destry (094HO13666), Gillette Final Cut (200HO03280), Comestar Lauthority (200HO05588), Carrousel Resurrect-Red (200HO09442), Pursuit September Storm (200HO03067), Comestar Stormatic (200HO04144), Ladino Park Talent (200HO07030), Gillette Windbrook (200HO03501), and Gillette Windhammer (250HO00914).

Serum cholesterol levels were analyzed using the MIXED procedure of SAS (v. 9.4; SAS Institute Inc., Cary, NC) with the following model:

$$\begin{array}{cc} y_{ijklmn} & =\mu +CS_{i}+\sum_{j=1}^{2}b_{j}\times DIM^{j}(LS_{k})\\ & +DCC_{l}(PS_{m})+Cow_{n}(CS_{i})+{ɛ}_{ijklmn,} \end{array}$$

where *y_ijklmn_* = serum cholesterol concentration (mg/dL); *μ* = mean; *CS* = carrier status *i* (carrier or control); *b_j_* = coefficient of regression on DIM and DIM^2^ nested within lactating status *k* (*LS*; dry cow/heifer or lactating cow); *DCC_l_* = days carried calf nested within pregnancy status *m* (*PS*; pregnant or not pregnant); *Cow_n_* = the random effect of individual cow nested within carrier status; and *ɛ* = random error. Least squares means were generated using a Tukey adjustment, with DIM and DCC set to 0. Cholesterol increased from calving to approximately 200 DIM by 105 mg/dL and then declined slowly; in contrast, cholesterol decreased over the progression of gestation (−23 mg/dL).

Daily phenotypes of milking time, milk conductivity, and activity of CD carriers and controls were analyzed using the MIXED procedure:

$$\begin{array}{cc} y_{ijklmn} & =\mu +CS_{i}+DIM_{j}(LG_{k})\\ & +Date_{l}+Cow_{m}\times lact_{n}(CS_{i})+{ɛ}_{ijklmn,} \end{array}$$

where *y_ijklmn_* = dependent variable of milking time, milk conductivity, or activity; *μ* = mean; *CS* = carrier status *i* (carrier or control); *DIM* = days in milk grouped in 2-wk intervals *j* (1 to ≥22) nested within lactation group (*LG*) *k* (1, 2, ≥3); *Date* = random effect of date of measurement *l*; *Cow* = the random effect of individual cow *m* in lactation number *n* nested within carrier status; and *ɛ* = random error. Least squares means were generated using a Tukey adjustment. The model used to analyze daily BW and daily milk yields was the same except that regression on genomic PTA of either BW composite (BW analysis) or milk yield (milk yield analysis) was included.

For the elite show cow analysis, chi-squared tests for equal proportions were conducted using the stats package in R (2019; https://www.R-project.org/) to determine whether the proportions of CD versus noncarrier daughters of the eligible sires differed from equality. Four tests were performed to determine whether show ring success was associated with CD carrier status: placing in the top 10 with cows allowed to contribute multiple years, placing in the top 5 with cows allowed to contribute multiple years, top 10 cows only considering individuals once, and top 5 cows only considering individuals once.

Table 1 displays results from serum cholesterol concentration and farm record analyses. On average, serum cholesterol concentrations of CD carriers were 26.06 mg/dL lower than those of controls (*P* < 0.001). The intraclass correlation coefficient of the model was 0.3355 for animals with repeated measures of serum cholesterol. Figure 1 displays a boxplot of serum cholesterol (mg/dL) across the carrier and control groups. Serum cholesterol concentration of CD carriers ranged from 61.3 to 208.8 mg/dL with a median value of 116.4 mg/dL, whereas control animal concentrations ranged from 69.1 to 260.5 mg/dL with a median of 131.7 mg/dL. Higher serum cholesterol in noncarriers is supported by the results of Kipp et al. (2016), Gross et al. (2016), Saleem et al. (2016), and Jacinto et al. (2019). However, our average serum cholesterol falls high on the reference range of 80 to 120 mg/dL and above the reported averages of controls and carriers from previous studies (Jacinto et al., 2019). This could be due to the kit used for serum analysis or due to an environmental effect because all animals were part of the same herd.

**Table 1:** Results of analysis of phenotypic traits between cholesterol deficiency (CD) carriers and controls for serum cholesterol concentration, milk yield, milking time, milk conductivity, activity, and BW

Dependent variable	Status	LSM estimate	SE	Difference	SE	Adjusted *P*-value
Cholesterol^1^ (mg/dL)	CD	90.15	7.49	−26.06	7.26	0.0007***
	Control	116.21	6.45			
Milk yield (kg)	CD	38.33	0.86	0.89	1.16	0.4447
	Control	37.44	0.79			
Milking time (min:s)	CD	05:11	00:14	−00:57	00:19	0.0039**
	Control	06:08	00:13			
Milk conductivity (mS)	CD	9.72	0.08	0.05	0.10	0.6731
	Control	9.67	0.07			
Activity (steps/h)	CD	109.58	7.41	4.99	9.90	0.6160
	Control	104.59	6.82			
BW (kg)	CD	620.90	7.30	−20.62	10.04	0.0437*
	Control	641.52	6.61			

1 Serum cholesterol concentration was the only analysis that included animals without lactation data.

* *P* < 0.05

** *P* < 0.01

*** *P* < 0.001.

**Figure 1:**
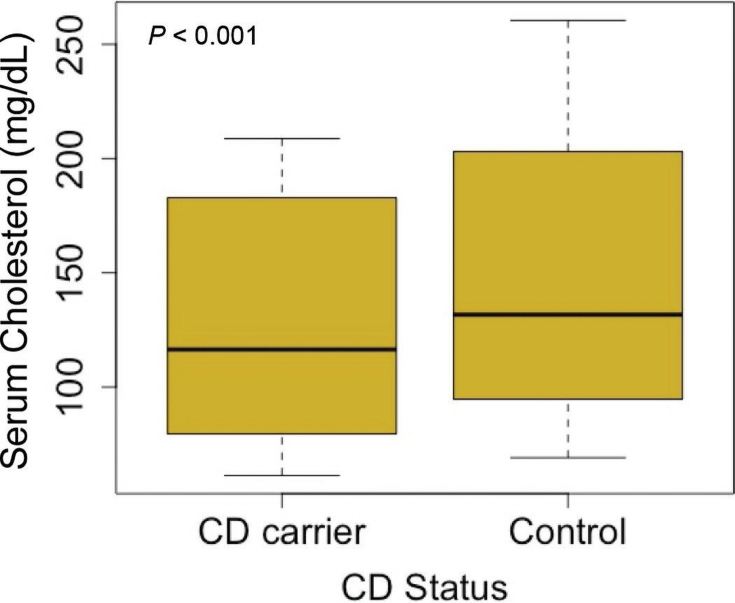
Serum cholesterol concentrations of cholesterol deficiency (CD) carriers and controls. The box spans from quartile 1 to quartile 3; the heavy line indicates the median serum cholesterol concentration; and the whiskers span from the minimum and maximum serum cholesterol concentration.

We found no significant differences between lactating CD carriers and controls in daily milk yield, milk conductivity, or daily activity (*P* > 0.05). Lactating CD carriers were, on average, 33.44 kg lighter than controls (*P* < 0.05) and milked out about 1 min faster than controls (*P* < 0.01). Cholesterol deficiency status was significantly associated with lower daily BW, even after accounting for genomic PTA of BW composite (every additional point of genomic PTA was associated with an increase of 24.01 ± 6.46 kg in daily BW). It has yet to be determined if this is because carriers carry less fat or because they were smaller in frame size and stature. Carriers of CD could carry less condition as it is known that higher circulating APOB levels are positively correlated with central fat distribution in humans (Okosun et al., 1999). Stunted growth is a symptom of calves with the homozygous *APOB* mutation and was also observed by Häfliger et al. (2019) in clinically affected heterozygotes. However, caution is warranted with these results because of the relatively limited number of cows in our data. Likewise, additional investigation in milking speed of CD carriers should be investigated in a larger population before further speculations of causality can be made.

The results of the chi-squared analysis are shown in Table 2. There were 231 instances where a daughter of a CD carrier sire placed in the top 10 of her class at World Dairy Expo; of those instances, 129 were CD carriers and 102 were noncarriers, which showed a tendency to differ (*P* = 0.08) from an expected frequency of 115.5 CD carriers and 115.5 non-CD carriers if CD status is unrelated to show ring success. Because some daughters were shown and placed in the top 10 multiple times, the 231 instances represented results from 135 daughters of the CD carrier sires; of these, 93 were Braedale Goldwyn daughters. If each daughter was only counted once, there were 72 carriers and 63 noncarriers, which did not differ significantly from expectation. In the top 5 placings, there were 135 instances (82 CD carriers and 55 noncarriers) comprised of 84 individual cows (48 CD carriers and 36 noncarriers). Carriers were more heavily represented in the top 5 of their class and significantly (*P* < 0.05) more than noncarriers when repeated wins were considered. Cholesterol is one of the main lipids of bovine skin, and “thin, loose, and pliable” skin is preferred in type evaluations (Rabinowitz and Shapiro, 1972; PDCA, 2009). However, skin thickness and other reasons that CD could contribute to show ring success are highly speculative. It is apparent that CD alone does not account for the success of daughters from bulls such as Braedale Goldwyn because noncarrier paternal siblings were also successful. Nevertheless, it is clear that being a carrier of CD did not negatively affect the potential for success in the country's most elite Holstein show and may even provide some benefit.

**Table 2:** Counts of daughters of cholesterol deficiency (CD)-carrying sires that placed in the top 10 or 5 in cow classes at World Dairy Expo from the years 2006 to 2019 allowing for individual animals to be counted for winning in multiple years and counting individual animals only once

Class	CD carrier	Noncarrier	Total	*P*-value
Top 10: Repeat placings	129	102	231	0.076†
Top 10: Individuals	72	63	135	0.439
Top 5: Repeat placings	82	55	137	0.0211*
Top 5: Individuals	48	36	84	0.1904

† *P* < 0.10

* *P* < 0.05.

Serum cholesterol levels of cattle that carry CD were lower than those of their noncarrier herdmates. Carriers also had faster milking speeds and lower BW than their herdmates, but results were based on a limited sample of carriers. Elite show cows that carry the *APOB* mutation are not at a disadvantage when evaluated for type and may carry an advantage in elite type competitions.
